# Audit of Parenteral Nutrition Use in Surgical Patients: Compliance With the 2017 American Society for Parenteral and Enteral Nutrition Guidelines

**DOI:** 10.7759/cureus.114218

**Published:** 2026-08-09

**Authors:** Telagathoti Anvesh, TP Elamurugan, Sarath Chandra Sistla

**Affiliations:** 1 Surgery, Jawaharlal Institute of Postgraduate Medical Education and Research, Puducherry, IND

**Keywords:** appropriateness of parenteral nutrition, aspen guidelines, bowel surgery, central line-associated bloodstream infection (clabsi), clinical audit, nutrition and metabolism, parenteral nutrition (pn), refeeding syndrome (rs), surgical nutrition, total parenteral nutrition (tpn)

## Abstract

Background

Prospective data on parenteral nutrition (PN) practices in surgical patients from India are limited. Auditing PN use with respect to indications, administration patterns, vascular access, monitoring, and complications provides insight into current clinical practice.

Objectives

To evaluate the adherence of PN practices at our institution to the 2017 American Society for Parenteral and Enteral Nutrition (ASPEN) guideline recommendations.

Methods

A hospital-based prospective observational study was conducted in the department of general surgery between April 2019 and April 2020. Adult patients who received PN and provided informed consent were enrolled consecutively. Data on patient demographics, indications for PN, patterns of use, vascular access, monitoring practices, complications, and clinical outcomes were collected prospectively from case records, the hospital information system (HIS), and the picture archiving and communication system (PACS). Categorical variables were summarized as frequencies and percentages, whereas continuous variables were summarized as the mean ± standard deviation or median with interquartile range (IQR), as appropriate. PN practices were assessed against the 2017 ASPEN guideline recommendations.

Results

Forty-seven patients met the inclusion criteria. The most common indications for PN were postoperative anastomotic leak (11 patients) and enterocutaneous fistula (10 patients). The median PN duration was seven days (IQR, 4.5-11.5 days). Before PN initiation, the central venous catheter tip position was radiologically confirmed in 40 patients, and 43 patients underwent risk assessment for refeeding syndrome. Daily monitoring of the catheter insertion site, vital signs, and fluid intake and output was performed, whereas body weight and nutritional requirements were reassessed weekly. Dysglycemia occurred in 34 patients, and blood glucose monitoring was less frequent than recommended by the ASPEN guidelines. Hyponatremia (78.72%), hypomagnesemia (78.72%), and hyperglycemia (72.34%) were the most common metabolic complications. Catheter-related bloodstream infection occurred in 11 patients, fungemia in three, and pneumothorax in one.

Conclusion

PN practices at our institution demonstrated substantial adherence to the 2017 ASPEN guideline recommendations. However, important gaps remained in central venous catheter care documentation and blood glucose monitoring, highlighting opportunities to improve the quality and safety of PN delivery through enhanced documentation and adherence to guideline-recommended monitoring practices.

## Introduction

Nutrition is defined as the science of food in relation to health and disease [1]. It plays a vital role in maintaining the structural and functional integrity of the body [2]. In surgical patients, the hypermetabolic response to surgical stress disrupts homeostasis and increases metabolic demands [3]. Rapid depletion of hepatic glycogen stores is followed by skeletal muscle protein breakdown to support hepatic gluconeogenesis, resulting in loss of lean body mass [4]. This catabolic response is associated with increased perioperative morbidity [5].

Preoperative malnutrition is associated with increased postoperative morbidity and mortality in surgical patients [6]. According to the National Confidential Enquiry into Patient Outcome and Death (NCEPOD) audit, 19-60% of surgical patients are malnourished [7]. In these patients, perioperative nutritional assessment and appropriate nutritional support can attenuate the stress response and improve surgical outcomes [4]. Although enteral nutrition is the preferred route of nutritional support, parenteral nutrition (PN) is indicated when the gastrointestinal tract is inaccessible or non-functional [8]. PN is defined as a method of nutritional delivery that bypasses the gastrointestinal tract and provides key nutrients intravenously, such as amino acids, glucose, lipids, vitamins, and minerals [6]. Despite being a life-saving intervention, PN is associated with potentially serious complications [7,9].

Safe PN administration requires accurate nutritional assessment, appropriate formulation of the PN regimen, secure vascular access, and regular clinical and biochemical monitoring. Previous studies, including those by NCEPOD and Dyson et al., have reported deficiencies in PN practice [7,9]. Although Ramakrishnan et al. evaluated PN use in an Indian setting, PN-related complications were not assessed [10]. Therefore, this prospective clinical audit aimed to evaluate the indications, patterns of use, vascular access practices, monitoring practices, and complications associated with PN in adult surgical patients at a tertiary care center in India and to assess adherence to the 2017 American Society for Parenteral and Enteral Nutrition (ASPEN) guideline recommendations [11].

## Materials and methods

Study design

This prospective observational clinical audit was conducted among patients admitted to the Department of General Surgery, Jawaharlal Institute of Postgraduate Medical Education and Research (JIPMER), Puducherry, between April 2019 and April 2020. As this was a clinical audit, all eligible patients receiving PN during the study period were identified prospectively and enrolled consecutively after providing written informed consent. No formal sample size calculation was performed. The study protocol was approved by the Institutional Ethics Committee, JIPMER.

Inclusion and exclusion criteria

All adult inpatients admitted to the Department of General Surgery who received PN during the study period and provided informed consent were included in the study. Patients who received PN but did not provide informed consent were excluded from the study.

Data collection

All participants were informed about the study in their native language, and their questions were addressed before enrollment. Written informed consent was obtained from all participants prior to inclusion in the study.

Demographic, clinical, biochemical, and outcome data were prospectively collected by the principal investigator using a structured, investigator-designed data collection form based on the study objectives and the 2017 ASPEN guideline recommendations [11]. The variables collected included age, sex, underlying diagnosis, comorbidities, surgical status, indications for PN, duration and type of PN, enteral feeding trial, dietitian involvement, body weight, body mass index (BMI), calculation of caloric and protein requirements, PN initiation, vascular access technique, central venous catheter (CVC) tip confirmation, blood glucose, serum electrolytes, liver function tests, bilirubin, triglyceride levels, refeeding syndrome risk assessment, and PN-related complications.

Data were obtained from case records, discharge summaries, the hospital information system (HIS), and the picture archiving and communication system (PACS). Case records and discharge summaries were used to obtain demographic and clinical information, caloric and protein requirement calculations, and vascular access information. CVC tip confirmation was verified using PACS, whereas biochemical monitoring and PN-related complications were verified using HIS and PACS.

Operational definitions

Operational definitions of the study outcome variables were based on the 2017 ASPEN guideline recommendations and standard clinical definitions, where applicable [11]. The operational definitions used in this study are presented in Table 1.

**Table 1:** Operational definitions of study outcome variables. PN: parenteral nutrition; AST: aspartate aminotransferase; ALT: alanine aminotransferase; ALP: alkaline phosphatase; GGT: gamma-glutamyl transferase.

Variable	Operational definition
Hyperglycemia	Blood glucose >180 mg/dL
Hypoglycemia	Blood glucose <70 mg/dL
Hypertriglyceridemia	Serum triglycerides >200 mg/dL
Hyponatremia	Serum sodium <130 mEq/L
Hypernatremia	Serum sodium >150 mEq/L
Hypokalemia	Serum potassium <3.0 mEq/L
Hyperkalemia	Serum potassium >5.5 mEq/L
Hypomagnesemia	Serum magnesium <1.3 mEq/L
Hypermagnesemia	Serum magnesium >2.5 mEq/L
Hypocalcemia	Serum calcium <9 mg/dL
Hypercalcemia	Serum calcium >11 mg/dL
Hypophosphatemia	Serum phosphorus <2.0 mg/dL
Hyperphosphatemia	Serum phosphorus >5.0 mg/dL
Refeeding syndrome	Concurrent occurrence of hypokalemia, hypomagnesemia, and hypophosphatemia following initiation of PN
Parenteral nutrition associated liver disease (PNALD)	Serum AST >35 IU/L, ALT >35 IU/L, ALP >120 IU/L, GGT >55 IU/L, and direct bilirubin >2 mg/dL
Central line-associated bloodstream infection (CLABSI)	Isolation of the same pathogen from central and peripheral blood cultures, with the central blood culture becoming positive ≥2 hours earlier
Pneumothorax	Air in the pleural space confirmed by chest radiography following central venous catheter insertion
Fungemia	Isolation of a fungal pathogen from a central venous catheter blood sample during PN

Statistical analysis

Data were entered into Microsoft Excel (Microsoft Corporation, Redmond, WA) spreadsheets and analyzed using SPSS version 20.0 (IBM Corp., Armonk, NY). Continuous variables were summarized as the mean ± standard deviation (SD) or median with interquartile range (IQR), as appropriate. Categorical variables were summarized as frequencies and percentages.

The outcome measures of this study included indications for PN, patterns of PN use, vascular access practices, monitoring practices, and PN-related complications. The appropriateness of PN practice was assessed by comparing observed clinical practice across these predefined outcome measures with the 2017 ASPEN guideline recommendations [11]. PN practice was considered adherent when the observed clinical practice was consistent with the corresponding ASPEN recommendations for each assessed domain [11].

No missing data were identified for the predefined audit variables, as all required information was available from the case records, HIS, and PACS. Consequently, no statistical imputation was required. As this was a descriptive clinical audit, no inferential statistical analyses were performed.

## Results

A total of 47 patients met the inclusion criteria. All provided informed consent and were included in the study.

Table 2 shows the baseline demographic characteristics and the systems involved. The mean age of the study population was 46 ± 13 years, with the highest proportion of patients belonging to the 41-50-year age group. Among the study participants, 36 (76.60%) had no comorbidities. Five patients (10.64%) had type 2 diabetes mellitus, three (6.38%) had cerebrovascular disease, and three (6.38%) had coronary artery disease. Of the 47 patients, 35 (74.47%) underwent surgical intervention, while 12 (25.53%) were managed conservatively.

**Table 2:** Baseline demographic characteristics and systems involved. NSTI: necrotizing soft tissue infection.

Variable		Frequency (n)	Percentage (%)
Gender	Male	31	65.96%
	Female	16	34.04%
Comorbidities	No comorbidities	36	76.60%
	Type 2 diabetes mellitus	5	10.64%
	Cerebrovascular disease	3	6.38%
	Coronary artery disease	3	6.38%
System involved	Gastrointestinal tract	36	76.60%
	Hepatobiliary system	6	12.76%
	Pancreas	4	8.51%
	Skeletal system (NSTI with septic encephalopathy)	1	2.13%

Table 3 summarizes the indications for PN. Among the study population, 11 patients (23.40%) received PN for postoperative anastomotic leak, and 10 patients (21.27%) received PN for enterocutaneous fistula. Six patients (12.76%) received PN for postoperative paralytic ileus, and another six (12.76%) received PN for preoperative nutritional support. Four patients (8.51%) received PN for acute severe pancreatitis, three (6.38%) for gastric outlet obstruction, three (6.38%) for short bowel syndrome, one (2.13%) for efferent loop obstruction, one (2.13%) for small bowel obstruction, one (2.13%) for diabetic foot with necrotizing soft tissue infection (NSTI) and septic encephalopathy, and one (2.13%) for dysphagia secondary to carcinoma of the esophagus.

**Table 3:** Indications for parenteral nutrition.

Indication	Frequency (n)	Percentage (%)
Postoperative paralytic ileus	6	12.76
Preoperative nutritional support	6	12.76
Postoperative anastomotic leak	11	23.40
Enterocutaneous fistula	10	21.27
Small bowel obstruction	1	2.13
Gastric outlet obstruction	3	6.38
Severe acute pancreatitis	4	8.51
Short bowel syndrome	3	6.38
Efferent loop obstruction	1	2.13
Diabetic foot with necrotizing soft tissue infection (NSTI) with septic encephalopathy	1	2.13
			Dysphagia secondary to carcinoma of the esophagus	1	2.13

Of the 47 patients, 26 received PN for ≤7 days, 13 for 8-14 days, three for 15-21 days, and five for >21 days. The median duration of PN was seven days, with an IQR of 4.5-11.5 days.

Table 4 shows the pattern of PN use. Of the 47 patients, 20 (42.55%) initiated PN within three to five days of hospital admission, while 27 (57.45%) initiated PN after enteral nutrition was deemed not feasible. All patients received PN using multichamber bags. In all patients, a multilumen CVC was used.

**Table 4:** Patterns of parenteral nutrition (PN) use.

Parameter	Category	Frequency	Percentage (%)
Enteral nutrition trial before PN	Yes	35	74.47
	No	12	25.53
Type of PN	Total parenteral nutrition	30	63.83
	Supplemental parenteral nutrition	17	36.17
Dietitian opinion before initiation of PN	Yes	28	59.57
	No	19	40.43
Assessment of weight before PN	Yes	47	100
	No	0	0
Assessment of BMI before PN	Yes	47	100
	No	0	0
Assessment of serum albumin levels before PN	Yes	43	91.49
	No	4	8.51
Calculation of nutritional requirements before PN therapy	Dietitian	28	59.57
	Resident	19	40.43
Assessment of risk factors for refeeding syndrome before PN	Yes	43	91.48
	No	4	8.51

In 46 patients, the CVC was inserted through the internal jugular vein, while in one patient, the subclavian vein was used. The CVC tip position was confirmed by chest X-ray in 39 patients and by high-resolution computed tomography (HRCT) of the thorax in one patient. In the remaining seven patients, no radiological confirmation of CVC tip position was documented. The vascular access site, vital signs, and fluid intake and output were monitored daily in all patients. Body weight, caloric requirements, and protein requirements were reassessed weekly.

Table 5 shows the monitoring practices for electrolytes and liver function tests (LFTs) during PN. Among the study population, electrolyte levels were monitored every 24 hours in 30 patients (63.83%) and every 48 hours in 17 patients (36.17%). The median interval for electrolyte monitoring was 24 hours (IQR, 21.6-26.4 hours).

**Table 5:** Monitoring of serum electrolytes and liver function tests during parenteral nutrition.

Parameter		Frequency (n)	Percentage (%)	Median	IQR
						Electrolytes	Once in 24 hours	30	63.82	24 hours	21.6-26.4 hours
Once in 48 hours	17	36.17									
Once in more than 72 hours	0	0									
			Liver function test	Not done	1		2.13	24 hours	17.76-30.24 hours		
Once in 72 hours	45	95.74									
Once in more than 72 hours	1	2.13							

LFTs were performed at intervals of no more than 72 hours in 45 patients (95.74%). The median interval for LFT monitoring was 24 hours (IQR, 17.76-30.24 hours).

Table 6 shows the monitoring practices for blood glucose during PN. After PN initiation, 34 of the 47 patients (72.34%) developed dysglycemia. In all 34 patients, blood glucose was monitored once daily.

**Table 6:** Blood glucose monitoring during parenteral nutrition

Parameter		Frequency	Percentage (%)	Median	IQR
						Patients with uncontrolled blood glucose (n = 34)	Once in 8 hours	0	0	24 hours	21.6-26.4 hours
Once in more than 8 hours	34	72.34									
			Patients with controlled blood glucose (n = 13)	Once in 24 hours	8							17.02
Once in 48 hours	5	10.64										
				Once in more than 72 hours	0		0				

Among the remaining 13 patients with controlled blood glucose, monitoring was performed once daily in eight patients and once every two days in five patients. The median interval for blood glucose monitoring was 24 hours (IQR, 21.6-26.4 hours).

Table 7 shows the catheter-related complications. Of the 47 patients, 32 had no catheter-related complications, 11 developed central line-associated bloodstream infection (CLABSI), three developed fungemia, and one developed pneumothorax.

**Table 7:** Central venous catheter-related complications.

Central line-related complications	Frequency	Percentage (%)
No complications	32	68.09
Central line-associated bloodstream infection (CLABSI)	11	23.40
Pneumothorax	1	2.13
			Fungemia	3	6.38

Following PN initiation, metabolic and nutritional complications were observed among the 47 patients. Hyperglycemia occurred in 34 patients (72.34%), hypoglycemia in 25 (53.19%), hyperkalemia in five (10.64%), hypokalemia in 31 (65.96%), hypernatremia in seven (14.89%), hyponatremia in 37 (78.72%), hypercalcemia in four (8.51%), hypocalcemia in 22 (46.81%), hypermagnesemia in five (10.64%), hypomagnesemia in 37 (78.72%), hyperphosphatemia in eight (17.02%), hypophosphatemia in 24 (51.06%), refeeding syndrome in 21 (44.68%), hypertriglyceridemia in 12 (25.53%), and parenteral nutrition-associated liver disease (PNALD) in 26 patients (55.32%). All metabolic and nutritional complications observed during PN were appropriately managed according to standard institutional clinical practice.

Documentation regarding PN indications, patterns of PN use, CVC insertion site, CVC type, insertion technique, monitoring parameters, complications, and operator designation was available in the case records of all patients. Although radiological confirmation of CVC tip position was available in PACS for 40 patients, documentation of CVC tip position in the clinical case records was absent. Of the 47 patients, 35 were discharged, while 12 died during hospitalization due to their underlying primary disease.

## Discussion

Malnutrition is common among hospitalized surgical patients, and PN is an essential therapeutic modality when enteral nutrition (EN) is not feasible. However, inappropriate PN use remains a significant clinical concern [7,12,13]. Given the limited data on the Indian population [10], this prospective study evaluated PN use in adult surgical patients.

Among the study population, the highest proportion of participants belonged to the 41-50-year age group, with a mean age of 46 years (SD, 13), comparable to that reported by Ramakrishnan et al. (54 years; SD, 14.8 years) [10]. In our study, 31 (66%) patients were male, and 16 (34%) were female. A similar sex distribution was reported by Ramakrishnan et al., Chan et al., and Nardo et al. [10,14,15].

In this study, 36 patients (76.60%) who received PN had gastrointestinal pathology. Similar findings were observed by Kraft et al. (64.5%), Ramakrishnan et al. (55.6%), Nardo et al. (54%), and Chan et al. (46.3%) [10,14-16]. Postoperative anastomotic leak was the most common indication for PN, accounting for 11 (23.40%) patients. Similarly, Chan et al. found that postoperative complications were the leading indication for PN (51.5%), including paralytic ileus (37%) and anastomotic leak (14.5%) [14]. Dyson et al. also identified postoperative paralytic ileus as a common indication, accounting for 34.3% of patients receiving PN [9].

Among the study population, 46 (97.87%) patients received PN for an appropriate indication. One patient (2.13%) received PN despite having an intact and functional gastrointestinal tract. Although this did not meet the recommendations of the 2017 ASPEN guidelines, the patient had septic encephalopathy with altered sensorium and was at high risk of aspiration with enteral feeding [11]. Furthermore, the patient was deemed unfit to undergo sedation for percutaneous endoscopic gastrostomy (PEG) tube placement. Therefore, PN was considered the most appropriate option for nutritional support in this clinical context.

The duration of PN ranged from one to 66 days, with a median of seven days (IQR, 4.5-11.5 days). This was comparable to the findings of Dyson et al. [9] but shorter than those reported in the NCEPOD audit (eight days) and by Chan et al. (nine days) [7,14], and longer than that reported by Ramakrishnan et al. (six days) [10].

In accordance with the 2017 ASPEN guidelines, EN was attempted before PN initiation in 35 (74.47%) participants [11]. Dyson et al. documented a lower rate of EN trials (32%) before PN initiation [9]. Information on EN trials was unavailable in the studies by Ramakrishnan et al. and Kraft et al. [10,16].

Among the study population, 30 (63.83%) patients received PN as total parenteral nutrition (TPN), while 17 (36.17%) received supplemental parenteral nutrition (SPN). In contrast, Chan et al. used TPN exclusively in all participants (100%) [14]. The relatively higher use of SPN in our study may reflect greater tolerance of EN in 17 patients (36.17%), allowing PN to be provided as supplementary nutritional support.

At our institution, PN initiation, nutritional requirement calculations, and PN formulation were primarily performed by treating residents, with dietitian involvement when available.

In this study, a dietitian was involved in the management of 28 patients (59.57%), whereas 19 patients (40.43%) received PN without dietitian involvement. In the absence of a dietitian, the PN formulation was prescribed by the treating resident. Comparable findings were reported by Dyson et al. and in the NCEPOD audit, where dietitian involvement was documented in approximately 50% of patients [7,9]. Although the 2017 ASPEN guidelines recommend dietitian involvement in the assessment, prescription, and monitoring of PN, dietitians were not available outside regular working hours at our institution [11]. As the majority of patients in this study required emergency surgical care, PN was frequently initiated during periods when dietitians were unavailable. Therefore, the relatively low rate of dietitian involvement was likely attributable to the absence of an on-call dietitian service.

The 2017 ASPEN guidelines recommend assessing the risk of refeeding syndrome (RS) before initiating PN [11]. In our study, RS risk was assessed in 43 patients (91.48%), while four (8.52%) did not undergo assessment. Comparable findings were reported in the NCEPOD audit, where 86% of patients underwent RS risk assessment [7].

In this study, 20 (42.55%) patients initiated PN within three to five days of hospital admission, including six with postoperative paralytic ileus. This was consistent with the 2017 ASPEN guideline recommendation for initiating PN within three to five days in malnourished patients or those at nutritional risk who are unable to tolerate EN [11].

In this study, 27 (57.45%) patients initiated PN within 24 hours of developing EN intolerance. As these patients were malnourished or at risk of malnutrition, this practice was consistent with the 2017 ASPEN guideline recommendations [11].

All patients received PN using premixed multichamber bags because alternative formulations, such as two-in-one admixtures, were unavailable at our institution. In patients receiving SPN, the remaining nutritional requirements were met through the oral or enteral route, whereas those receiving TPN received additional intravenous amino acid solution (Aminocore) or 25% dextrose as required.

All patients received PN through a CVC, with the internal jugular vein used in 46 (97.87%) patients and the subclavian vein in one (2.13%). Similar findings were reported by Chan et al., who observed CVC use in 99.21% of patients receiving PN [14].

Among the study population, CVC tip position was radiologically confirmed in 40 (85.10%) patients. Although documentation of the CVC insertion site, catheter type, insertion technique, and operator designation was complete, CVC tip position was not documented in any of the case records.

In accordance with the 2017 ASPEN guidelines, the vascular access site, vital signs, fluid intake and output, and catheter-related parameters were monitored daily. Body weight was measured using a weighing scale, while caloric and protein requirements were calculated using weight-based methods and reassessed weekly [11].

Among the study population, serum electrolytes were monitored every 24 hours in 30 (63.82%) patients and every 48 hours in 17 (36.17%). The median monitoring interval was 24 hours (IQR, 21.6-26.4 hours). A comparable 24-hour monitoring interval was reported by Dyson et al. and in the NCEPOD audit [7,9]. In contrast, the frequency of electrolyte monitoring was not reported by Ramakrishnan et al. or Chan et al. [10,14].

Among the study population, 34 patients (72.34%) developed dysglycemia. The 2017 ASPEN guidelines recommend monitoring blood glucose at least every eight hours in patients with dysglycemia receiving PN [11]. In our study, monitoring was less frequent than recommended, with all patients undergoing blood glucose monitoring only once every 24 hours. A similar pattern of once-daily monitoring was reported by DeLegge et al. [17].

Among the 13 (27.66%) patients with controlled blood glucose, monitoring was performed every 24 hours in eight (17.02%) patients and every 48 hours in five (10.63%) patients. The median monitoring interval was 24 hours (IQR, 21.6-26.4 hours).

Among the study population, catheter-related complications occurred in 15 (31.91%) patients. In comparison, Dyson et al. reported a catheter-related complication rate of 15% [9]. The higher rate observed in this study may reflect differences in patient characteristics, illness severity, or catheter management practices.

Although evidence of overnutrition was observed in some patients, the metabolic abnormalities cannot be attributed solely to PN, as underlying illness, surgery, and other clinical factors may also have contributed.

RS occurred in 21 (44.68%) patients; however, no deaths were attributable to this complication. The observational design precludes conclusions regarding the effect of risk assessment or management on this outcome.

Overall, PN practice at our institution demonstrated substantial adherence to the 2017 ASPEN guidelines [11]. However, deficiencies were identified in CVC care documentation, dietitian involvement, and blood glucose monitoring. These findings were communicated to the nutrition support team and surgical units, with reinforcement of guideline-based monitoring and standardized documentation practices to improve patient safety.

The main limitations of this study were its relatively small sample size and single-center design, which may limit the generalizability of the findings. As this was a clinical audit of all eligible patients during the study period, no formal sample size calculation was performed. The descriptive nature of the study precluded the identification of predictors of inappropriate PN use or PN-related complications, and reliance on medical records may have introduced documentation bias. As an observational audit of PN practices and guideline adherence, the study did not include a comparator group, limiting outcome comparisons with non-PN or EN groups. In addition, because this audit was conducted between 2019 and 2020, the findings reflect institutional practice during the audit period rather than current practice.

Despite these limitations, this prospective clinical audit provides valuable data on PN practices in adult surgical patients from an Indian tertiary care center, where published evidence remains limited. The study systematically assessed multiple domains of PN practice against the 2017 ASPEN guideline recommendations, providing a benchmark for future audits and quality improvement initiatives [11].

## Conclusions

PN practice at our institution demonstrated substantial adherence to the 2017 ASPEN guidelines, particularly with respect to indications for PN, patterns of PN use, vascular access techniques, and electrolyte monitoring.

However, important gaps were identified in blood glucose monitoring, CVC documentation, and dietitian involvement, indicating that adherence was not complete. These findings highlight opportunities for quality improvement through standardized documentation, improved access to dietetic services, adherence to recommended monitoring protocols, and regular clinical audits to optimize the quality and safety of PN practice.
